# Symptom Burden, Self-Efficacy, and Satisfaction with Nursing Care in Adults Undergoing Hemodialysis in Oman: A Cross-Sectional Study

**DOI:** 10.3390/nursrep16020065

**Published:** 2026-02-13

**Authors:** Eilean Rathinasamy Lazarus, Joshua Kanaabi Muliira, Jihad Hassan, Ramesh Chandrababu, Zakariya Al-Naamani, Ram Kumar Palani

**Affiliations:** 1 Department of Adult Health and Critical Care, College of Nursing, Sultan Qaboos University, Al Khoudh, Muscat 123, Oman; j.muliira@squ.edu.om (J.K.M.); j.hassan@squ.edu.om (J.H.); 2 Medical Research Center, Sultan Qaboos University, Al Khoudh, Muscat 123, Oman; 3 Department of Medical Surgical Nursing, Sri Ramachandra Faculty of Nursing, Sri Ramachandra Institute of Higher Education and Research (DU), Chennai 600116, India; ramesh.c@sriramachandra.edu.in; 4 Department of Fundamentals and Administration, College of Nursing, Sultan Qaboos University, Al Khoudh, Muscat 123, Oman; z.alnaamani3@squ.edu.om; 5 School of Health Sciences-Medicine, University of Georgia, Tbilisi 0171, Georgia; ramkumar.pe2002@gmail.com

**Keywords:** hemodialysis, symptom burden, patient satisfaction, nursing care, self-efficacy, chronic kidney disease, Oman, dialysis nursing, psychological symptoms, physical symptoms, nursing

## Abstract

**Background:** Adults on maintenance hemodialysis experience multiple physical and psychological symptoms that can affect confidence in self-management and perception of care received from healthcare providers. Understanding the interplay between symptom burden, self-management self-efficacy, and perceptions about care received is essential to inform patient-centered nephrology nursing. **Aim:** This cross-sectional study aimed to describe dialysis symptom burden, self-efficacy to manage chronic disease, and satisfaction with nursing care, and to examine associations among these variables in adults undergoing maintenance hemodialysis in Oman. **Methods:** A cross-sectional study using consecutive sampling was conducted among 232 adults on maintenance hemodialysis at two dialysis units in Muscat, Oman. Data were collected using the Dialysis Symptom Index, the nursing care satisfaction questionnaire, and the self-efficacy scale. Descriptive, correlation, and multivariable linear regression analysis were used to summarize the findings. **Results:** The mean age was 55.9 years and the most common comorbidities were diabetes (58.2%) and hypertension (74.1%). Symptom burden was substantial, with over half reporting muscle soreness, anxiety, sleep disturbance, dry mouth, pruritus, appetite loss, and dyspnea, although severity was generally mild–moderate (1.1–1.6/4). Satisfaction with nursing care was high (90.2%), while self-efficacy was moderate (mean 30.52/44). Patient satisfaction correlated positively with self-efficacy (r = 0.25, *p* < 0.001), but not with symptom burden (r = 0.08, *p* = 0.24); Self-efficacy showed a small positive correlation with dialysis symptom burden (r = 0.14, *p* = 0.03), suggesting that patients who were more aware of and reported more symptoms also perceived themselves as more actively engaged in managing their illness. In multivariable analysis, higher satisfaction and more favorable laboratory indicators independently predicted higher self-efficacy. **Conclusions:** Adults on hemodialysis reported high satisfaction with nursing care but continued to experience multiple physical and psychological symptoms and had only moderate self-efficacy to manage their condition. There is a need to integrate structured symptom assessment and targeted, nurse-led self-management support intervention into routine dialysis care to reduce symptom burden and enhance patients’ confidence in managing their illness.

## 1. Introduction

Chronic kidney disease (CKD) and end-stage kidney disease (ESKD) are both increasing across the world contributing significantly to the non-communicable disease burden, and implications for health systems globally [1,2,3,4,5,6]. Recent Global Burden of Disease analyses estimate that 674 to 788 million people were living with CKD in 2021–2023, a marked rise over the past three decades [2,3,5]. Globally, the age-standardized prevalence of CKD in adults is around 10–14%, and CKD accounted for roughly 1.4 million deaths and more than 40 million disability-adjusted life years (DALYs) in 2019 [2,3]. The number of people progressing to kidney failure requiring kidney replacement therapy (KFRT) has also risen steadily, and the prevalence of treated ESKD is projected to continue increasing as aging, diabetes, and hypertension grow in the population [1,3,4].

There are substantial disparities in access to kidney replacement therapy (KRT), with many individuals in low- and middle-income countries unable to receive dialysis or transplantation [1,3]. It is estimated that up to 50% of all people needing KRT worldwide may not receive it, and the highest proportion without access lives in low-income settings [1]. The inequities underscore the need to optimize availability, efficiency, and quality of dialysis care [1,3,4]. Within the Gulf Cooperation Council (GCC) region, dialysis prevalence is among the highest globally and continues to rise alongside the growing burden of diabetes and hypertension [1,4]. Regional data indicate that hemodialysis is the dominant KRT in GCC and Eastern Mediterranean countries, whereas peritoneal dialysis (PD) and conservative kidney management remain underutilized [1,3].

Oman is one of the countries in the GCC, and has experienced a pronounced increase in kidney failure and ESKD [6,7,8,9]. Oman has experienced a sustained increase in treated kidney failure over the past four decades, with national registry analyses showing progressive growth in the incidence and prevalence of patients receiving renal replacement therapy up to 2015 [6,7,10]. Historical registry data indicate that by 2015 the Ministry of Health operated 18 renal dialysis centers serving approximately 1439 patients on dialysis per year, with a mean annual prevalence of about 725 dialysis patients and a mean annual incidence of around 204 new kidney failure patients between 1983 and 2015 [6,7,10].

More recent regional data from the 2023 International Society of Nephrology (ISN) Global Kidney Health Atlas report an overall chronic dialysis prevalence of 350.5 patients per million population in the ISN Middle East region, with hemodialysis accounting for most treatment and peritoneal dialysis remaining underused [11,12]. Within this region, Oman is notable for having a national CKD registry, but it reports one of the lowest incidences of kidney transplantation (2.2 per million population), highlighting ongoing challenges in expanding transplant capacity despite a high and rising burden of kidney failure [13].

In Oman, ESKD is more common in males (57.1%) than females (42.9%), and the median age of incident is 53 years, commonly with comorbidities such as diabetic nephropathy (46%) and hypertensive nephropathy (19%) [6,9,11]. To meet this rising demand KRT, the Ministry of Health in Oman has substantially expanded dialysis services nationwide. By 2015, 18 renal dialysis centers were providing care to approximately 1439 patients on dialysis per annum, with a mean annual prevalence of about 725 dialysis patients and a mean annual incidence of 204 new kidney failure patients between 1983 and 2015 [7,13].

The financial impact of this expansion on the healthcare system is considerable, with the annual cost of dialysis care rising from roughly 2.25 million Omani rials (5.9 million USD) in 1998 to 9.54 million Omani rials (24.8 million USD) in 2015, a more than threefold increase [7]. Consequently, there is a strong policy and clinical imperative to ensure that dialysis care in Oman not only prolongs survival but also maximizes health-related quality of life and other patient-reported outcomes [6,7]. Despite the comprehensive epidemiologic descriptions of ESKD, less is known about the day-to-day symptom experiences, treatment burden, and psychosocial outcomes of Omani adults receiving maintenance hemodialysis [6,7,11]. Symptom burden and experiences can impact self-care, adherence to treatment, and self-efficacy for self-management. International studies show that adults on hemodialysis commonly report high symptom burden with emphasis on fatigue, pruritus, sleep disturbance, pain, and psychological distress, all of which can impair daily functioning and health-related quality of life, adherence to treatment, and self-management [14,15,16].

Nursing care is central to the delivery of hemodialysis and nurses spend the most time with patients, performing symptom assessment, education, psychosocial support, and other roles [8,16]. Moreover, high satisfaction with nursing care is associated with better communication about symptoms, stronger therapeutic relationships, and improved adherence to dialysis regimens [1,3]. Self-efficacy, defined as an individual’s confidence in their ability to perform behaviors necessary to manage a chronic condition, is a key modifiable determinant of self-care in dialysis patients [17,18]. Among adults on hemodialysis, higher self-efficacy has been linked to better adherence to fluid and dietary restrictions, engagement in treatment, and quality of life, whereas low self-efficacy can amplify the impact of symptoms and hinder effective coping with the demands of hemodialysis [19,20,21]. In addition to symptom assessment and self-management support, infection-prevention practices around central venous catheters are an important nursing-sensitive process of dialysis care; for example, a recent narrative review reported that hub devices (such as antimicrobial or disinfecting caps and connectors) were generally associated with lower rates of catheter-related bloodstream infections compared with standard solid caps in hemodialysis populations [22].

However, there is a paucity of empirical research that simultaneously examines dialysis symptom burden, self-efficacy, and satisfaction with nursing care in patients on hemodialysis in Oman, which limits the development of context-specific nursing interventions [6,7,9,12]. Therefore, this cross-sectional, observational study aimed to describe dialysis symptom burden, self-efficacy to manage chronic disease, and satisfaction with nursing care among adults undergoing maintenance hemodialysis in Oman, and to examine associations between these variables using multivariable linear regression analysis.

Based on prior literature, we hypothesized that higher dialysis symptom burden would be associated with lower self-efficacy and lower satisfaction with nursing care, whereas higher self-efficacy and higher satisfaction would each be associated with more favorable clinical indicators (e.g., nutritional and hematologic parameters). We also explored whether patient satisfaction with nursing care independently predicted self-efficacy after adjusting for socio-demographic and clinical variables.

## 2. Materials and Methods

### 2.1. Study Design

This study employed a quantitative cross-sectional design to examine dialysis symptom burden, self-efficacy, and satisfaction with nursing care among adults undergoing maintenance hemodialysis at two government dialysis units in Muscat, Oman. Data were collected at a single point in time using structured questionnaires and clinical record abstraction [19,23].

### 2.2. Study Population and Setting

The study population comprised Omani adult patients with ESKD receiving maintenance hemodialysis at Bausher Dialysis Unit and Seeb Dialysis Unit in Muscat, Oman. These two units were purposively selected because they are the largest government hemodialysis centers in the country, providing services to a diverse catchment of patients and thereby enhancing the representativeness of the sample in terms of sociodemographic and clinical characteristics. Eligible participants were Omani adults aged 18 years or older, receiving maintenance hemodialysis for at least 3 months at Bausher or Seeb Dialysis Units, clinically stable (no acute illness or hospitalization in the preceding month), and able to communicate in Arabic or English to provide informed consent. Patients with documented cognitive impairment, severe psychiatric illness, or significant hearing/visual limitations were excluded to ensure valid self-reported data.

### 2.3. Sample Size and Sampling Technique

The sample size was 232 adults undergoing maintenance hemodialysis at Bausher and Seeb Dialysis Units in Muscat, Oman. The minimum required sample was initially estimated using a single-proportion formula for cross-sectional studies. Assuming an anticipated prevalence of dialysis symptom burden of approximately 50% (to maximize sample size), a 95% confidence level, and a 5% margin of error, this yielded a target sample size of 196. Considering an estimated 15–20% possible non-response rate, the final sample was adjusted to 232. The sample of 232 exceeds the required minimum, thereby improving the precision of prevalence estimates and the power to detect associations between symptom burden, self-efficacy, and satisfaction with nursing care.

A consecutive sampling technique was used, whereby all eligible patients attending routine hemodialysis sessions at the two units during the data collection period were invited to participate until the required sample size was reached. Consecutive sampling is commonly used in hemodialysis research because it is practical in unit-based settings, minimizes selection bias compared to convenience sampling, and allows inclusion of a broad mix of patients with varying sociodemographic and clinical profiles.

### 2.4. Instruments Used for Data Collection

Data were collected using previously validated Arabic-language versions of standardized instruments. The scales included a socio-demographic and clinical data form, the Dialysis Symptom Index (DSI) to assess dialysis-related symptom burden, the Self-Efficacy to Manage Chronic Disease 6-Item Scale (SEMCD-6) to measure patients’ confidence in managing their illness, and the Patient Satisfaction with Nursing Care Quality Questionnaire (PSNCQQ) to evaluate satisfaction with nursing care. These Arabic versions had been translated, culturally adapted, and psychometrically tested in prior studies, demonstrating acceptable reliability and validity in Arabic-speaking patient populations, and were adopted in the present study without further modification.

The dialysis symptom burden was assessed using the Arabic version of the DSI, a 30-item instrument that evaluates the presence and severity of common physical and psychological symptoms on a Likert-type scale, with higher scores indicating greater symptom burden [24]. Previous studies have reported Cronbach’s alpha values of about 0.82–0.91 for the total DSI score, indicating good to excellent internal consistency [25,26]. In the current study the Arabic DSI showed excellent reliability, with a Cronbach’s alpha of 0.80.

Self-efficacy was assessed using the Arabic version of the SEMCD-6 [27]. This instrument rates patients’ confidence in managing fatigue, physical discomfort, emotional distress, and other consequences of chronic illness on an 11-point scale from 0 (“not at all confident”) to 10 (“totally confident”), with higher mean scores reflecting greater self-efficacy [27,28]. The original scale showed excellent internal consistency (Cronbach’s alpha = 0.91) and has been successfully adapted into several languages and chronic disease groups, including a European Portuguese version with Cronbach’s alpha of 0.89 [28]. In the hemodialysis populations, the SEMCD-6 has been used to examine the link between symptom burden, self-efficacy, and quality of life [19]. In the current study, the SEMCD-6 (Arabic version) demonstrated good internal consistency (Cronbach’s alpha = 0.72).

Patient satisfaction with nursing care was measured using the Arabic PSNCQQ, which has demonstrated good content validity and high internal consistency in Arabic-speaking hospital populations [29,30]. The PSNCQQ includes Likert-type items on information and instructions, nurses’ concern and responsiveness, involvement of family, coordination and continuity of care, and overall nursing care, with higher scores indicating higher satisfaction [29]. The global health item (‘In general, would you say your health is…’) is part of the original PSNCQQ and was included in the total satisfaction score as per instrument scoring guidelines. In the current study the PSNCQQ demonstrated a Cronbach’s alpha of 0.75.

### 2.5. Data Collection Procedure

Data were collected between September 2023 and March 2024 during routine hemodialysis sessions at the two dialysis units. Eligible patients were approached during treatment, informed about the study, and those who agreed provided written informed consent before participation. The survey was mainly self-administered and took approximately 20–25 min to complete. Most questionnaires were completed by participants themselves; however, for those with visual, literacy, or physical limitations, trained research assistants read items aloud in Arabic and recorded responses verbatim, following a standardized script, avoiding leading explanations, and providing only neutral clarifications of item wording to minimize interviewer influence. Clinical and laboratory data (e.g., comorbidities, dialysis vintage, blood urea nitrogen, creatinine, hemoglobin, albumin, calcium, Kt/V) were obtained from medical records using a standardized abstraction sheet. To ensure data quality, the team was trained in eligibility screening, informed consent procedures, standardized questionnaire administration, and chart abstraction, and completed questionnaires were checked immediately for missing or inconsistent responses; all data were anonymized, coded, and stored in electronic files with restricted access.

### 2.6. Data Management and Missing Data

Completed questionnaires were checked for missing or inconsistent responses, and unclear items were clarified with participants when possible. Across the 232 participants, missing data on the main study variables were rare (all key variables had less than 5% missing values. For multi-item scales, if more than 20% of items were missing, the scale score was set to missing and excluded from scale-level analyses; when 20% or fewer items were missing, missing values were imputed using the respondent’s mean for completed items on that scale. Descriptive item-level analyses used available-case data without further imputation. For correlation and regression analyses, we used complete-case analysis, including only participants with non-missing data on the variables entered in each model.

Data were entered and analyzed using IBM SPSS Statistics, version 28.0. Scale scores for symptom burden, satisfaction with nursing care, and self-efficacy were computed per instrument guidelines, and internal consistency was assessed using Cronbach’s alpha. Descriptive statistics (frequencies, percentages, means, standard deviations) summarized participant characteristics and main study variables. Pearson correlation coefficients were used to examine bivariate associations between total symptom burden, self-efficacy, and satisfaction scores; for satisfaction, we inspected histograms, skewness, and kurtosis, and also calculated Spearman rank correlations as a sensitivity analysis given the high mean and limited dispersion. Multivariable linear regression was then performed with total self-efficacy (SEMCD-6) as the dependent variable and symptom burden (DSI), satisfaction (PSNCQQ), and selected sociodemographic, clinical, and laboratory variables as predictors. Covariates were retained if they showed an association with self-efficacy at *p* < 0.10 in bivariate analyses or were considered theoretically important (e.g., age, sex, education, employment, dialysis vintage, diabetes, hemoglobin, albumin, Kt/V). Linearity, normality, and homoscedasticity of residuals were assessed using standard diagnostic plots, and variance inflation factors indicated no problematic multicollinearity. A *p* value < 0.05 was considered statistically significant.

At the bivariate level, we report correlation coefficients with 95% confidence intervals to quantify the precision and magnitude of associations, in addition to *p* values. For the multivariable model, unstandardized regression coefficients and their 95% confidence intervals are presented to facilitate clinical interpretation of effect sizes.

### 2.7. Ethical Considerations

The study adhered to established ethical standards for research involving human participants. Approval was obtained from the Research and Ethics Committee of the College of Nursing at Sultan Qaboos University (SQU-EC/382/2023 dated 5 January 2023) and from the Ministry of Health’s Research and Ethics Committee in Oman (MoH/CSR/23/28853 dated 16 January 2023). All eligible patients were informed about the study objectives, procedures, potential risks and benefits, and their right to withdraw at any time without affecting their care before providing written informed consent. Confidentiality and anonymity were ensured by assigning unique identification codes to participants, omitting personal identifiers from the dataset, and storing all study documents and electronic files in password-protected and access-restricted locations. Data were used solely for research purposes, and findings are reported in aggregates.

## 3. Results

### 3.1. Characteristics of the Participants

The sample comprised 232 adults receiving maintenance hemodialysis, with a mean age of 55.85 ± 13.25 years. Males constituted a slight majority of the sample (57.8%), and most participants were married (61.6%), had had primary education (33.2%), were unemployed (58.2%), and had been living with kidney disease for more than three years (59.5%), indicating a predominantly long-standing illness trajectory. Comorbidities were common, with hypertension (74.1%) and diabetes mellitus (58.2%) being the most frequently reported conditions, and about one-third having coronary artery disease (34.1%). Most patients had been on hemodialysis for 4–12 months (84.9%), and nearly two-thirds received dialysis three times per week (65.5%). The mean biochemical values reflected typical hemodialysis profiles, with a mean BUN of 59.93 ± 14.46 mg/dL, creatinine 9.05 ± 1.95 mg/dL, hemoglobin 10.67 ± 1.51 g/dL, and a mean Kt/V of 1.42 ± 0.31, indicating generally adequate dialysis dose.

We examined differences in dialysis symptom burden, self-efficacy, and satisfaction with nursing care across key sociodemographic and clinical subgroups. Mean symptom burden did not differ significantly by sex or marital status (*p* > 0.05), whereas participants with lower education or longer dialysis vintage tended to report slightly higher symptom scores (where *p* < 0.05). Self-efficacy scores were significantly higher among employed participants and those without diabetes, while satisfaction with nursing care remained consistently high across subgroups, with no statistically significant differences by age or sex (*p* > 0.05) (Table 1).

### 3.2. Dialysis Symptom Burden

Dialysis symptom burden was substantial, with several symptoms affecting more than half of participants. The highest-ranking symptoms by prevalence were muscle soreness (64.2%), feeling anxious (62.1%), trouble falling asleep (60.3%), dry mouth (59.5%), itching (57.3%), decreased appetite (57.3%), trouble staying asleep (56.9%), and shortness of breath (56.5%). Slightly lower, but still prominent, were feeling nervous (53.4%), bone/joint pain (52.6%), headache (51.7%), and difficulty concentrating (50.0%).

In terms of severity, the symptoms with comparatively higher mean scores included dry mouth (1.59 ± 1.55), muscle soreness (1.56 ± 1.47), feeling anxious (1.56 ± 1.52), and shortness of breath (mean = 1.54 ± 1.62). By contrast, symptoms such as diarrhea, decrease in sex interest, feeling sad, and difficulty becoming sexually aroused were lower-ranking in both prevalence (32–34%) and severity (≤1.0). Symptoms with the highest prevalence included muscle soreness, anxiety, sleep disturbance, dry mouth, pruritus, decreased appetite, and shortness of breath, while diarrhea, decreased sexual interest, feeling sad, and difficulty becoming sexually aroused showed lower prevalence and severity as depicted in Table 2.

### 3.3. Self-Efficacy to Manage Chronic Disease

Self-efficacy to manage chronic disease was moderate among the sample. The mean total self-efficacy score was 30.52 ± 4.82 on a possible range of 17–44, indicating an intermediate level of confidence in managing disease-related problems. Item means ranged from 4.91 to 5.23 on the 0–10 scale, suggesting that many patients felt only moderately confident across different self-management domains. Participants reported similar confidence in controlling fatigue (mean = 4.91 ± 1.56), physical discomfort or pain (mean = 5.08 ± 1.51), emotional distress (mean = 4.98 ± 1.70), and other symptoms or health problems (mean = 5.23 ± 1.48) to avoid interference with desired activities. Confidence in performing tasks and activities needed to manage the health condition and reduce the need to see a doctor (mean = 5.23 ± 1.61), and in using strategies beyond medication to limit the illness’s impact on daily life (mean = 5.00 ± 1.65), was also moderate, highlighting a need to enhance patients’ self-management skills through tailored interventions as depicted in Table 3.

### 3.4. Patient Satisfaction with Nursing Care Quality

Patient satisfaction with nursing care quality was high, with a mean total satisfaction score of 90.19 ± 4.02 (range 79–100). Most item means were rated in the range of 3.8–4.0 on the 5-point scale, indicating consistent positive perceptions of information, interpersonal care, and coordination. Higher ratings were observed for information given by nurses (mean = 4.00 ± 0.73), informing family or friends (mean = 4.02 ± 0.80), concern and caring by nurses (mean = 3.95 ± 0.79), attention to patients’ condition (mean = 3.97 ± 0.77), and coordination of care (mean = 3.96 ± 0.79). The distribution of total satisfaction scores was left-skewed, with most patients clustering at the upper end of the scale (mean 90.19 ± 4.02 on a 0–100 scale; observed range 79–100), indicating a marked ceiling effect and limited variability. In sensitivity analyses using Spearman correlations, the direction and significance of the associations between satisfaction, self-efficacy, and symptom burden were similar to those obtained with Pearson coefficients, although effect sizes were small, consistent with the restricted range of satisfaction scores. Overall quality of care, overall quality of nursing care, and willingness to recommend the hospital were also rated favorably. Relatively lower, though still positive, scores were noted for nursing staff response to calls (mean = 3.81 ± 0.81), privacy (mean = 3.84 ± 0.81), and coordination of care after discharge (mean = 3.83 ± 0.84), suggesting specific areas where nursing services could be further strengthened as depicted in Table 4.

### 3.5. Relationship Between Symptom Burden, Self-Efficacy, and Satisfaction with Nursing Care

Correlation analysis (Table 5) showed a small but statistically significant positive association between patient satisfaction and self-efficacy (Pearson r = 0.25, *p* < 0.001), indicating that participants who reported higher satisfaction with nursing care also tended to report higher confidence in managing their chronic illness. In contrast, there was no significant correlation between patient satisfaction and dialysis symptom burden (r = 0.08, *p* = 0.24). Self-efficacy demonstrated a small positive correlation with dialysis symptom burden (r = 0.14, *p* = 0.03); although statistically significant, the small magnitude indicates only a modest relationship and suggests that higher confidence in self-management did not necessarily translate into lower reported symptom load in this cohort. To account for the skewed, ceiling-affected distribution of satisfaction scores, we repeated these analyses using Spearman’s rank correlation, which yielded very similar estimates and significance levels (e.g., satisfaction–self-efficacy ρ ≈ 0.24, *p* < 0.001), supporting the robustness of the findings to non-normality and restricted range.

The multivariable linear regression model predicting self-efficacy was statistically significant (F(20, 211) = 13.13, *p* < 0.001)) and explained a substantial proportion of the variance in self-efficacy scores (R^2^ = 0.67; adjusted R^2^ = 0.64). Higher patient satisfaction with nursing care remained a positive predictor of self-efficacy (B = 0.15, β = 0.13, *p* = 0.003; 95% CI 0.05–0.25), such that each one-point increase in satisfaction was associated with a 0.15-point increase in self-efficacy after adjustment for sociodemographic, clinical, and laboratory variables. Retired participants had significantly lower self-efficacy compared with the reference occupational group (B = −4.49, β = −0.09, *p* = 0.04; 95% CI −8.85 to −0.14). More favorable laboratory indicators, including higher BUN, creatinine, calcium, albumin, hemoglobin, and hematocrit, were also associated with higher self-efficacy (all *p* < 0.001), with 95% CIs for B ranging from 0.08 to 0.13 for BUN, 0.51 to 0.94 for creatinine, 1.50 to 2.28 for calcium, 2.18 to 3.90 for albumin, 0.85 to 1.38 for hemoglobin, and 0.26 to 0.45 for hematocrit. Other sociodemographic and clinical covariates entered in the model were not significantly associated with self-efficacy in the fully adjusted model (Table 6) (Supplementary Material Appendix S2. Analytic procedures in IBM SPSS Statistics).

For nurses, these findings underscore the importance of coupling strong therapeutic relationships and technical care with systematic symptom enquiry and tailored coaching to build patients’ confidence in managing fatigue, sleep disturbance, pain, and psychological distress, rather than assuming that high satisfaction alone reflects adequate symptom control.

## 4. Discussion

Adults on hemodialysis in Oman in this study reported a predominantly mild–moderate symptom burden, moderate self-efficacy, and high satisfaction with nursing care, a pattern broadly aligned with international dialysis data [14,15,16,19,21,31,32,33,34,35,36]. The very high mean satisfaction score (90.2%) and consistently favorable ratings across communication, attentiveness, coordination, and overall nursing care parallel reports from Egypt and other countries where patients are particularly positive about nurse-related aspects of care [31,32,33,34]. For example, Helmy et al. found that Egyptian hemodialysis patients were generally satisfied with dialysis care and most satisfied with nurse-related aspects, including monitoring and technical competence. Likewise, international surveys have documented high satisfaction with nursing staff and the dialysis environment even when other dimensions of care, such as physician communication or system-level factors, are rated less favorably [32,33,34].

The high satisfaction observed in the present Omani cohort may reflect strong nurse–patient relationships in relatively stable, unit-based care, culturally valued interpersonal support, adequate nurse–patient ratios in government facilities, and free access to dialysis services [6,7,8,11,12,13,31,33]. Slightly lower scores for privacy, response to calls, and post-discharge coordination mirror gaps reported elsewhere, where structural constraints and understaffing limit nurses’ ability to provide individualized privacy and follow-up, despite high ratings for interpersonal care. Because our sample was drawn from two large, publicly funded government units that provide dialysis free at the point of delivery and excluded recently hospitalized patients, the high satisfaction levels may not fully reflect experiences in smaller or private centers or among patients with more unstable disease.

The sample experienced a high prevalence of dialysis-related symptoms, with sleep disturbance, musculoskeletal discomfort, pruritus, dry mouth, appetite loss, dyspnea, and anxiety each affecting more than half of participants, although mean severities clustered in the mild–moderate range [14,15,16,19,24,35,36]. This pattern is consistent with multi-center DSI and KDIGO-aligned work showing that patients commonly report multiple concurrent symptoms—especially fatigue, sleep problems, pruritus, pain, and psychological distress—with overall severity often modest but cumulatively burdensome. Qualitative research from Oman further highlights fatigue as a dominant and complex symptom, with patients describing profound physical and mental tiredness and using a range of self-devised strategies to cope with its impact on daily life and quality of life [8,9,10,11]. Similar symptom profiles, including prominent sleep disturbance, musculoskeletal pain, and gastrointestinal symptoms, have been described in South Asia and other low- and middle-income settings. The similarity in core symptom clusters across settings underscores the intrinsic symptomatology of maintenance hemodialysis, whereas the relatively low severity scores in this sample may reflect adequate dialysis dose, relatively short dialysis vintage for many participants, and possible under-reporting due to cultural norms around expressing distress [6,7,8,11,13,35,36,37].

Self-efficacy scores in this study were moderate, with item means around the midpoint of the 0–10 scale, indicating that many patients felt only partly confident in controlling fatigue, pain, emotional distress, and other symptoms or tasks related to managing their condition. Similar intermediate self-efficacy levels have been observed in hemodialysis cohorts from Iran, Turkey, and other countries, where patients report moderate confidence in self-care and a need for structured educational and behavioral support. The positive association between patient satisfaction and self-efficacy, and the finding that satisfaction independently predicted self-efficacy after adjustment for socio-demographic and clinical variables, are consistent with evidence that supportive, responsive dialysis care and positive nurse–patient interactions enhance patients’ sense of control and readiness to engage in self-management behaviors [17,18,19,31,38].

The observed positive association between self-efficacy and laboratory indicators such as albumin, hemoglobin, and hematocrit suggests that patients with more favorable nutritional and hematologic profiles tend to report greater confidence in managing their condition; however, these cross-sectional relationships are exploratory and should not be interpreted as evidence of a causal or clinically ‘ideal’ level of these markers. This aligns with studies linking higher self-efficacy to better adherence to diet, fluid, and medication regimens and to improved quality of life in hemodialysis populations [17,18,19,20,21,38]. However, the cross-sectional design precludes conclusions about directionality; better physical health may enhance perceived self-efficacy, higher self-efficacy may support more effective self-care and improved clinical status, or both processes may operate in a bidirectional manner. By contrast, the small positive correlation between self-efficacy and dialysis symptom burden differs from studies where higher self-efficacy has been associated with lower symptom burden and better quality of life. One explanation is that more self-efficacious patients may be more aware of and willing to report symptoms, or that greater engagement with care leads to closer monitoring and recognition of symptomatology, inflating reported burden without necessarily reflecting poorer control. Unmeasured psychosocial factors such as resilience and depression, which are known to interact with self-efficacy and symptom perception, may also have influenced these relationships [37,38].

The finding that satisfaction with nursing care was not significantly correlated with overall symptom burden, despite high satisfaction scores and substantial symptom prevalence, is consistent with international work showing that patients can rate relational and technical aspects of dialysis care favorably even when they experience multiple ongoing symptoms [39,40]. Large surveys across Europe and South America, and registry-based studies, report high ratings for nurses’ caring and technical competence alongside persistent symptom burden and dissatisfaction with information provision [39,40,41]. In the present study, high satisfaction may reflect appreciation of nurses’ efforts within structural and disease-related constraints, while symptom burden is driven predominantly by underlying disease and treatment factors rather than perceived nursing care quality. Taken together, these results highlight the importance of integrating structured symptom assessment and nurse-led self-management support into routine dialysis practice, recognizing that even highly satisfied patients may live with considerable symptom load and only moderate confidence in managing their condition [14,15,16,19,21,35,36,42,43]. They also point to an opportunity for targeted, nurse-led interventions—such as exercise, education, and psychosocial support—to improve both symptom control and self-efficacy in adults undergoing maintenance hemodialysis in Oman [14,15,16,20,21,31,35,42,43,44]. Although multicollinearity diagnostics suggested that correlations among laboratory variables such as hemoglobin and hematocrit did not reach problematic levels, the inclusion of multiple related biomarkers and the modest sample size mean that the regression findings should be interpreted cautiously as exploratory rather than confirmatory [19,21,22,35,36,37,38]. When developing structured protocols for dialysis nursing care, device-management steps around central venous catheters should also be standardized; for example, a recent bench study comparing different sealing caps for short-term dialysis catheters showed that no cap configuration eliminated blood backflow and that clamping consistently reduced backflow, suggesting that clamping should be routine practice when needle-free connectors are used [45].

### 4.1. Strength and Limitation of the Study

This cross-sectional study has several strengths. It included a relatively large sample of 232 adults on maintenance hemodialysis from two major government units in Muscat and simultaneously assessed symptom burden, satisfaction with nursing care, and self-efficacy using validated Arabic instruments and standardized clinical and laboratory data, allowing a multidimensional, nursing-focused appraisal of patients’ experiences in a setting where such evidence remains limited. By reporting effect sizes with confidence intervals rather than relying solely on *p* values, we aimed to support a more refined clinical interpretation of the strength and precision of the observed associations.

However, important limitations should be acknowledged. The cross-sectional design precludes causal inference between self-efficacy, symptom burden, satisfaction, and biochemical markers, and bidirectional effects cannot be excluded. Consecutive sampling of clinically stable patients in two large government units likely introduced selection bias towards a somewhat ‘healthier’ and more satisfied cohort and limits generalizability to smaller regional or private centers. Self-report measures may be affected by recall and social desirability bias, and cultural norms could have contributed to under-reporting of symptoms and overestimation of satisfaction. Because a minority of participants required assisted questionnaire completion, some degree of interviewer influence on responses cannot be entirely excluded, although standardized administration procedures were used to minimize this risk. Finally, the point-in-time laboratory data and potential residual collinearity among related biomarkers (e.g., hemoglobin and hematocrit), together with the absence of qualitative data and direct measures of psychosocial factors, mean that the regression findings—particularly the associations with biochemical markers—should be interpreted cautiously and as hypothesis-generating rather than as evidence of causal or clinically ‘optimal’ levels.

### 4.2. Implication for Clinical Practice

The high prevalence of sleep disturbance, musculoskeletal discomfort, pruritus, dry mouth, appetite loss, dyspnea, and anxiety in this cohort suggests that nurses should integrate brief, structured symptom assessments (for example, using the DSI) into routine hemodialysis care to systematically identify and prioritize these common problems for targeted management. Given that overall satisfaction with nursing care was high but scores were relatively lower for privacy, response to calls, and post-discharge coordination, unit-level quality improvement efforts should focus on practical strategies to enhance privacy (e.g., use of curtains and private discussions), timeliness of responses to patients’ requests, and structured discharge or follow-up education. As vascular access care is a core dialysis nursing responsibility, routine assessment and meticulous management of arteriovenous fistulas, grafts, and catheters—including surveillance for infection, thrombosis, and dysfunction—should be combined with symptom monitoring to prevent access-related complications and support treatment adequacy. The finding of only moderate self-efficacy, together with its positive association with patient satisfaction and several laboratory markers, highlights the need for nurse-led self-management support interventions that provide education, behavioral coaching, and ongoing reinforcement to strengthen patients’ confidence in managing fatigue, pain, emotional distress, diet, and fluid restrictions. Finally, the lack of association between satisfaction with nursing care and overall symptom burden highlights that even highly satisfied patients may continue to experience substantial symptom load, underscoring the importance of embedding symptom management protocols alongside relational, technical, and vascular-access-related aspects of care. The study was not powered or designed to test a fully specified causal model; rather, the observed associations among symptom burden, satisfaction with nursing care, self-efficacy, and laboratory indicators should be interpreted as exploratory and hypothesis-generating for future longitudinal and interventional research.

## 5. Conclusions

Adults receiving maintenance hemodialysis at two major government units in Muscat reported substantial but predominantly mild–moderate dialysis-related symptoms, high satisfaction with nursing care, and only moderate self-efficacy to manage their condition. These findings indicate that, even in settings where nursing care is rated very positively, patients may continue to live with multiple ongoing symptoms and limited confidence in self-management. From a nephrology nursing perspective, the results underscore the need to prioritize systematic symptom enquiry and nurse-led support to strengthen patients’ self-efficacy, rather than relying solely on global satisfaction as an indicator of care quality. As a cross-sectional study conducted in two units in Oman, the findings should not be generalized beyond similar settings, but they provide context-specific, patient-centered evidence to inform future nursing practice and research in hemodialysis care. As an exploratory cross-sectional analysis, these findings generate hypotheses about how symptom burden, perceived nursing care, and self-efficacy intersect in hemodialysis practice and point to specific targets for future nurse-led interventions and longitudinal studies.

## Figures and Tables

**Table 1:** Socio-Demographic/Clinical Characteristics of the Participants (*n* = 232).

Demographic Characteristics			Frequency		Percentage (%)
Age in years (M = 55.85 ± 13.25)	18–28	3		1.3	
	29–39	24		10.3	
	40–49	53		22.8	
	50–59	52		22.4	
	60–70	62		26.7	
	>70	38		16.4	
Gender	Male	134		57.8	
	Female	98		42.2	
Marital status	Married	143		61.6	
	Unmarried	42		18.1	
	Widow or divorced	47		20.2	
Education	No formal education	61		26.3	
	Primary education	77		33.2	
	High school education	64		27.6	
	Higher secondary school	9		3.9	
	Graduates	21		9.1	
Clinical Characteristics					
Occupation	Self employed	24		10.3	
	Government employee	55		23.7	
	Unemployed	135		58.2	
	Private employee	16		6.9	
	Retired	2		0.9	
Duration of illness in years	0–1	31		13.4	
	1–3	63		27.2	
	>3	138		59.5	
Comorbidities	Diabetes Mellitus	135		58.2	
	Hypertension	172		74.1	
	Coronary Artery Disease	79		34.1	
	Cancer	8		3.4	
	Dyslipidemia	6		2.6	
	Heart Failure	5		2.2	
	Sickle cell	1		0.4	
	SLE	2		0.9	
	Thalassemia	3		1.3	
Duration of hemodialysis treatment in months	4–12	197		84.9	
	>12	35		15.1	
Frequency of hemodialysis per week	Once	49		21.1	
	Twice	31		13.4	
	Thrice	152		65.5	
Laboratory Biomarker		Mean		SD	
Laboratory biomarker	BUN (mg/dL)	59.93		14.46	
	Creatinine (mg/dL)	9.05		1.95	
	Calcium (g/dL)	8.65		1.01	
	Albumin (g/dL)	3.86		0.47	
	Hemoglobin (g/dL)	10.67		1.51	
	Hematocrit (%)	31.45		4.45	
	Kt/V	1.42		0.31	

BUN—Blood Urea Nitrogen; SD—Standard Deviation; Kt/V—urea clearance (K), dialysis time (t), and the patient’s urea distribution volume (V, approximating total body water). SLE, Systemic Lupus Erythematous.

**Table 2:** Prevalence and Severity of Dialysis Symptoms among the study participants (*n* = 232).

Symptoms	Prevalence Frequency (%)	Severity Mean (SD)
Muscle soreness	149 (64.2%)	1.56 (1.47)
Feeling anxious	144 (62.1%)	1.56 (1.52)
Trouble falling asleep	140 (60.3%)	1.48 (1.51)
Dry mouth	138 (59.5%)	1.59 (1.55)
Itching	133 (57.3%)	1.36 (1.45)
Decreased appetite	133 (57.3%)	1.39 (1.49)
Trouble staying asleep	132 (56.9%)	1.47 (1.54)
Shortness of breath	131 (56.5%)	1.54 (1.62)
Feeling nervous	124 (53.4%)	1.42 (1.59)
Bone/joint pain	122 (52.6%)	1.42 (1.56)
Headache	120 (51.7%)	1.21 (1.40)
Difficulty concentrating	116 (50.0%)	1.33 (1.56)
Feeling tired/lack of energy	115 (49.6%)	1.11 (1.36)
Constipation	115 (49.5%)	1.26 (1.53)
Swelling in legs	114 (49.1%)	1.21 (1.48)
Muscle cramps	112 (48.3%)	1.17 (1.45)
Numbness/tingling in feet	111 (47.8%)	1.21 (1.48)
Light-headed/dizziness	104 (44.8%)	1.16 (1.49)
Vomiting	103 (44.3%)	1.09 (1.43)
Chest pain	100 (43.1%)	1.07 (1.41)
Restless legs	98 (42.2%)	1.05 (1.44)
Dry skin	94 (40.5%)	0.96 (1.37)
Cough	93 (40.1%)	1.06 (1.47)
Worrying	93 (40.1%)	1.03 (1.46)
Irritability	91 (39.2%)	1.00 (1.46)
Decrease in sex interest	89 (38.4%)	0.91 (1.36)
Feeling sad	84 (36.2%)	0.90 (1.37)
Nausea	106 (45.7%)	1.16 (1.46)
Diarrhea	78 (33.6%)	0.79 (1.28)
Difficulty becoming sexually aroused	75 (32.3%)	0.88 (1.44)

**Table 3:** Self-Efficacy to Manage Chronic Disease among the study participants (*n* = 232).

Items Measuring Self-Efficacy to Manage Chronic Disease	Mean	SD	Min	Max
How confident do you feel that you can keep the fatigue caused by your disease from interfering with the things you want to do?	4.91	1.56	1	10
How confident do you feel that you can keep the physical discomfort or pain of your disease from interfering with the things you want to do?	5.08	1.51	1	9
How confident do you feel that you can keep the emotional distress caused by your disease from interfering with the things you want to do?	4.98	1.70	1	10
How confident do you feel that you can keep any other symptoms or health problems you have from interfering with the things you want to do?	5.23	1.48	2	9
How confident do you feel that you can do the different tasks and activities needed to manage your health condition so as to reduce your need to see a doctor?	5.23	1.61	1	9
How confident do you feel that you can do things other than just taking medication to reduce how much your illness affects your everyday life?	5	1.65	1	9
Self-Efficacy Total	30.52	4.82	17	44

SD—Standard Deviation; Min—Minimum; Max—Maximum. Min and Max indicate the minimum and maximum values observed in the sample. Items are rated on a 0–10 scale, with higher scores indicating greater self-efficacy. The total self-efficacy score is calculated as the sum of all item scores.

**Table 4:** Patient Satisfaction with Nursing Care Quality among the study participants (*n* = 232).

	Mean	SD	Min	Max
Information you were given	3.91	0.74	2	5
Instructions	3.91	0.81	1	5
Ease of getting information	3.89	0.80	2	5
Information given by nurses	4	0.73	2	5
Informing family or friends	4.02	0.80	2	5
Involving family or friends in your care	3.88	0.78	2	5
Concern and caring by nurses	3.95	0.79	2	5
Attention of nurses to your condition	3.97	0.77	2	5
Recognition of your opinions	3.94	0.80	2	5
Consideration of your needs	3.94	0.78	2	5
The daily routine of the nurses	3.91	0.78	2	5
Helpfulness	3.96	0.788	2	5
Nursing staff response to your calls	3.81	0.81	2	5
Skill and competence of nurses	3.87	0.84	2	5
Coordination of care	3.96	0.79	2	5
Restful atmosphere provided by nurses	3.96	0.79	2	5
Privacy	3.84	0.81	2	5
Discharge instructions	3.91	0.80	2	5
Coordination of care after discharge	3.83	0.84	1	5
Overall quality of care and services	3.89	0.86	2	5
Overall quality of nursing care	3.89	0.82	1	5
In general, would you say your health is	3.97	0.79	2	5
Based on the nursing care I received, I would recommend this hospital	3.91	0.74	2	5
Satisfaction Total	90.19	4.02	79	100

SD—Standard Deviation; Min—Minimum; Max—Maximum.

**Table 5:** Correlations among Patient Satisfaction, Self-Efficacy, and Dialysis Symptom Burden (*n* = 232).

Variable	Patient Satisfaction	Self-Efficacy	Dialysis Symptom Burden
Patient satisfaction	1.00	0.25 (*p* = 0.001)	0.08 (*p* = 0.24)
Self-efficacy	0.25 (*p* = 0.001)	1.00	0.14 (*p* = 0.03)
Dialysis symptom burden	0.08 (*p* = 0.24)	0.14 (*p* = 0.03)	1.00

Values represent Pearson correlation coefficients (r); two-tailed p values are shown in parentheses. Diagonal elements represent correlations of each variable with itself (r = 1.00).

**Table 6:** Predictors of self-efficacy in multivariable linear regression (*n* = 232).

Predictor	B	Beta	*p* Value	95% CI
Patient satisfaction (total score)	0.15	0.13	0.003	0.05–0.25
Retired (vs. reference occupation)	−4.49	−0.09	0.04	−8.85–−0.14
BUN (mg/dL)	0.11	0.32	0.001	0.08–0.13
Creatinine (mg/dL)	0.72	0.29	0.001	0.51–0.94
Calcium (g/dL)	1.89	0.40	0.001	1.50–2.28
Albumin (g/dL)	3.04	0.30	0.001	2.18–3.90
Hemoglobin (g/dL)	1.11	0.35	0.001	0.85–1.38
Hematocrit (%)	0.36	0.33	0.001	0.26–0.45

Multivariable linear regression analysis of predictors of self-efficacy. B indicates the unstandardized regression coefficient, representing the change in the outcome per one-unit change in the predictor. β indicates the standardized regression coefficient, representing the association in standard deviation units. CI denotes the 95% confidence interval for B.

## Data Availability

Data are available on reasonable request from the corresponding author. The datasets are not publicly available due to institutional and national regulations on patient data, but de-identified data may be shared for non-commercial research purposes subject to ethics committee and institutional approvals, and completion of a data-sharing agreement when required.

## Supplementary Materials

The following supporting information can be downloaded at: https://www.mdpi.com/article/10.3390/nursrep16020065/s1, Appendix S1: STROBE Statement—Checklist of items that should be included in reports of cross-sectional studies; Appendix S2: Analytic procedures in IBM SPSS Statistics.

## Public Involvement Statement

There was no public involvement in any aspect of this research.

## Guidelines and Standards Statement

This manuscript was drafted in accordance with the Strengthening the Reporting of Observational Studies in Epidemiology (STROBE) Statement (cross-sectional checklist—Appendix S1) for observational cross-sectional research.

## Abbreviations

The following abbreviations are used in this manuscript:

BUN	Blood Urea Nitrogen
CI	Confidence Interval
CKD	Chronic Kidney Disease
DALYs	Disability-Adjusted Life Years
DSI	Dialysis Symptom Index
ECP	Effective Clinical Practice (journal title context)
ESKD	End-Stage Kidney Disease
GCC	Gulf Cooperation Council
KRT	Kidney Replacement Therapy
KFRT	Kidney Failure Requiring Replacement Therapy
Kt/V	Urea clearance (K), dialysis time (t), and distribution volume (V)
MoH	Ministry of Health (Oman)
PD	Peritoneal Dialysis
PSNCQQ	Patient Satisfaction with Nursing Care Quality Questionnaire
SD	Standard Deviation
SEMCD-6	Self-Efficacy to Manage Chronic Disease 6-Item Scale
SLE	Systemic Lupus Erythematosus (spelled “Erythematous” in text)
SPSS	Statistical Package for the Social Sciences (IBM SPSS Statistics)

## References

[1] Thurlow J.S., Joshi M., Yan G., Norris K.C., Agodoa L.Y., Yuan C.M., Nee R. (2021). Global epidemiology of end-stage kidney disease and disparities in kidney replacement therapy. Am. J. Nephrol..

[2] Deng L., Guo S., Liu Y., Zhou Y., Liu Y., Zheng X., Yu X., Shuai P. (2025). Global, regional, and national burden of chronic kidney disease and its underlying etiologies from 1990 to 2021: A systematic analysis for the Global Burden of Disease Study 2021. BMC Public Health.

[3] Jager K.J., Kovesdy C., Langham R., Rosenberg M., Jha V., Zoccali C. (2019). A single number for advocacy and communication—worldwide more than 850 million individuals have kidney diseases. Nephrol. Dial. Transplant..

[4] Rafferty Q., Stafford L.K., Vos T., Thomé F.S., Aalruz H., Abate Y.H., Abbafati C., ElHafeez S.A., Abedi A., Abiodun O.O. (2025). Global, regional, and national prevalence of kidney failure with replacement therapy and associated aetiologies, 1990–2023: A systematic analysis for the Global Burden of Disease Study 2023. Lancet Glob. Health.

[5] Francis A., Harhay M.N., Ong A.C.M., Tummalapalli S.L., Ortiz A., Fogo A.B., Fliser D., Roy-Chaudhury P., Fontana M., Nangaku M. (2024). Chronic kidney disease and the global public health agenda: An international consensus. Nat. Rev. Nephrol..

[6] Al Alawi I., Al Salmi I., Al Mawali A., Al Maimani Y., Sayer J.A. (2017). End-stage kidney failure in Oman: An analysis of registry data with an emphasis on congenital and inherited renal diseases. Int. J. Nephrol..

[7] Al Salmi I., Kamble P., Lazarus E.R., D’Souza M.S., Al Maimani Y., Hannawi S. (2021). Kidney disease-specific quality of life among patients on hemodialysis. Int. J. Nephrol..

[8] Al Rahbi I., Lazarus E.R., AlZaabi O., Al-Noumani H. (2025). Effectiveness of intradialytic muscle stretching exercise on muscle cramps among patients undergoing maintenance hemodialysis in Oman: A randomized controlled trial. J. Health Sci..

[9] Al-Naamani Z., Gormley K., Noble H., Santin O., Al Omari O., Al-Noumani H., Madkhali N. (2024). The lived experiences of fatigue among patients receiving haemodialysis in Oman: A qualitative exploration. BMC Nephrol..

[10] Al-Naamani Z., Gormley K., Noble H., Santin O., Al Omari O., Al-Noumani H., Madkhali N. (2025). Navigating strategies used to manage fatigue by patients undergoing hemodialysis: A qualitative exploratory design. BMC Nephrol..

[11] Al Balushi N., Al-Rawajfah O., Lazarus E.R., Al daken L., Al zaabi O. (2024). The effect of intradialytic exercise on biophysiological parameters and quality of life among patients undergoing maintenance hemodialysis in Oman: A comparative observational study. Nurs. Midwifery Stud..

[12] Karam S., Amouzegar A., Alshamsi I.R., Al Ghamdi S.M.G., Anwar S., Ghnaimat M., Saeed B., Arruebo S., Bello A.K., Caskey F.J. (2024). Capacity for the management of kidney failure in the International Society of Nephrology Middle East region: Report from the 2023 ISN Global Kidney Health Atlas (ISN-GKHA). Kidney Int. Suppl..

[13] Al Salmi I., Al-Maymmani Y., Al-Riyami M., Al-Rajhi W., Al-Za’abi R., Al-Alawi I., Ali M., Mohammed E.A.R., Al Rahbi F. (2021). Nephrology in the Sultanate of Oman. Nephrology Worldwide.

[14] Alshammari B., Edison J.S., Alkubati S.A., Alrasheeday A.M., Albagawi B., Alharbi L.L., Motakef H.I., Alshammari L., Siam B.G.A.E.-R., Alharbi N.A. (2025). Effectiveness of exercise in reducing symptom burden among hemodialysis patients: A non-pharmacological intervention approach. Front. Public Health.

[15] Ford E., Stewart K., Garcia E., Sharma M., Whitlock R., Getachew R., Rossum K., Duhamel T.A., Verrelli M., Zacharias J. (2024). Randomized controlled trial of the effect of an exercise rehabilitation program on symptom burden in maintenance hemodialysis: A clinical research protocol. Can. J. Kidney Health Dis..

[16] Lazarus E.R. (2019). Effectiveness of education and exercise on quality of life among patients undergoing hemodialysis. Clin. Epidemiol. Glob. Health.

[17] Ahn J.W., Lee S.M., Seo Y.H. (2022). Factors associated with self-care behavior in patients with pre-dialysis or dialysis-dependent chronic kidney disease. PLoS ONE.

[18] Turan G.B., Karabulutlu C., Özer Z. (2025). Analysis of the relationship between self-efficacy, adherence with diet therapy and fluid control in patients receiving hemodialysis treatment: A structural equation analysis. Hemodial. Int..

[19] Attoun A.S., Abuhalima D., Al-Jabi S.W., Zyoud S.H. (2025). Impact of symptom burden clusters on self-efficacy and quality of life in hemodialysis patients: A multicenter cross-sectional study. Sci. Rep..

[20] Lazarus E.R., Deva Amirtharaj A., Jacob D., Chandrababu R., Isac C. (2020). The effects of an olive-oil massage on hemodialysis patients suffering from fatigue at a hemodialysis unit in southern India: A randomized controlled trial. J. Complement. Integr. Med..

[21] Hafezieh A., Dehghan M., Taebi M., Iranmanesh S. (2020). Self-management, self-efficacy and knowledge among patients under haemodialysis: A case in Iran. J. Res. Nurs..

[22] Fiorina E., Giustivi D., Gotti F., Akyüz E., Privitera D. (2025). The use of Hub Devices to reduce catheter-related infections in dialysis patients: A narrative review. J. Vasc. Access.

[23] Zhang N., Tu H., Xiong X., Guo T., Zhang X., Cheng T., Peng Y., Li W., Chen Y. (2025). Correlation between symptom clusters and self-management among maintenance haemodialysis patients: A cross-sectional study. J. Clin. Nurs..

[24] Widiasta A., Labiba A.N., Tarigan R. (2025). Differences in quality of life among children with end-stage kidney disease undergoing hemodialysis and peritoneal dialysis. Nephro-Urol. Mon..

[25] Weisbord S.D., Fried L.F., Arnold R.M., Rotondi A.J., Fine M.J., Levenson D.J., E Switzer G. (2004). Development of a symptom assessment instrument for chronic hemodialysis patients: The Dialysis Symptom Index. J. Pain Symptom Manag..

[26] Barros J.P., Fonseca J.A., Pinto R., Pratas J., Correia R.J.C. (2024). Cross-cultural validation of the Portuguese version of the Dialysis Symptom Index for haemodialysis patients. J. Res. Nurs..

[27] Zhao W.M., Zhu L., Zhu Y., Li X.L., Shi R., Pan H.F., Wang D.-G., Yuan L. (2025). Increased dialysis symptom index burden in maintenance hemodialysis patients during the COVID-19 lockdown period. Ann. Med..

[28] Lorig K.R., Sobel D.S., Ritter P.L., Laurent D., Hobbs M. (2001). Effect of a self-management program on patients with chronic disease. Eff. Clin. Pract..

[29] Marconcin P., Tomé G., Carnide F., Yázigi F., Campos P., Pais S., Espanha M. (2021). Translation, cultural adaptation and validation of the Self-Efficacy to Manage Chronic Disease 6-Item Scale for European Portuguese. Acta Reumatol. Port..

[30] Albashayreh A., Al-Rawajfah O.M., Al-Awaisi H., Karkada S., Al Sabei S.D. (2019). Psychometric properties of an Arabic version of the Patient Satisfaction With Nursing Care Quality Questionnaire. J. Nurs. Res..

[31] Obaidi A., Rahmani A., Khader Y., Negarandeh R. (2025). Patients’ satisfaction with nursing care quality and its related factors: A cross-sectional study. Florence Nightingale J. Nurs..

[32] Helmy N.H., Hussein A., Kamal M., El Minshawy O., Wahsh E.A. (2022). Hemodialysis patients’ satisfaction with dialysis care: A cross-sectional prospective study conducted in a non-profitable care facility, Minia, Egypt. BMC Nephrol..

[33] Giannake G., Economou A., Metaxas T., Geitona M. (2023). Medical tourism in the region of Thessaly, Greece: Opinions and perspectives from healthcare providers. Sustainability.

[34] Palmer S.C., de Berardis G., Craig J.C., Tong A., Tonelli M., Pellegrini F., Ruospo M., Hegbrant J., Wollheim C., Celia E. (2014). Patient satisfaction with in-centre haemodialysis care: An international survey. BMJ Open.

[35] Yıldız A., Şahan S., Korkmaz E. (2023). Determination of nursing care perceptions of hemodialysis patients. Prog. Health Sci..

[36] You A.S., Kalantar S.S., Norris K.C., Peralta R.A., Narasaki Y., Fischman R., Fischman M., Semerjian A., Nakata T., Azadbadi Z. (2022). Dialysis symptom index burden and symptom clusters in a prospective cohort of dialysis patients. J. Nephrol..

[37] Mehrotra R., Davison S.N., Farrington K., Flythe J.E., Foo M., Madero M., Morton R.L., Tsukamoto Y., Unruh M.L., Cheung M. (2023). Managing the symptom burden associated with maintenance dialysis: Conclusions from a Kidney Disease: Improving Global Outcomes (KDIGO) Controversies Conference. Kidney Int..

[38] Pakaya R.E., Syam Y., Syahrul S. (2021). Correlation of self-efficacy and self-care of patients undergoing hemodialysis with their quality of life. Enferm. Clin..

[39] Al Nuairi A., Bermamet H., Abdulla H., Simsekler M.C.E., Anwar S., Lentine K.L. (2022). Identifying patient satisfaction determinants in hemodialysis settings: A systematic review. Risk Manag. Healthc. Policy.

[40] Tsanasidis M., Kafkia T., Papoutsis D., Kourakos M. (2025). Resilience, pain self-efficacy and health-related quality of life in Greek hemodialysis patients: A cross-sectional study. Int. J. Prev. Med..

[41] Sułkowski Ł., Matyja A., Matyja M. (2024). Social support and quality of life in hemodialysis patients: A comparative study with healthy controls. Medicina.

[42] Guerraoui A., Haesebaert J., Subtil F., Hanf W., Pelletier C., Jullien P., Villar E., Mezaache S., Filancia A., Fauvel J.-P. (2025). Concordance of symptoms perceived by patients receiving haemodialysis and those reported by nurses and nephrologists: A cross-sectional, multicentre, observational study using the REIN registry. Clin. Kidney J..

[43] Zheng X., Yang Z., Xu L., Wang A. (2025). Insights into the complexities of symptom management for hemodialysis patients: A systematic review of qualitative studies. Health Psychol. Rev..

[44] Foy M.D., Mang S., Mitchell A. (2023). End-stage renal disease and hemodialysis: Improving nursing care and patient experience. Nursing.

[45] Privitera D., Giustivi D., Langer T., Fiorina E., Gotti F., Rossini M., Brunoni B., Capsoni N., Dal Molin A., Zadek F. (2025). Effect of different sealing caps on the backflow of short-term dialysis catheters: A bench study. J. Vasc. Access.

