# Supplementary material for: Symptom Burden, Self-Efficacy, and Satisfaction with Nursing Care in Adults Undergoing Hemodialysis in Oman: A Cross-Sectional Study
Source: Nurs Rep. 2026 Feb 13;16(2):65. doi: 10.3390/nursrep16020065 (PMC12943693; doi:10.3390/nursrep16020065)
Supplement: Supplementary file 1 [file nursrep-16-00065-s001.zip › nursrep-4115554-supplementary.pdf]

Appendix S1 - STROBE Statement—Checklist of items that should be included in reports of ***cross-sectional studies***

	Item No	Recommendation	Page No.
**Title and abstract**	1	(*a*) Indicate the study's design with a commonly used term in the title or the abstract	1
		(*b*) Provide in the abstract an informative and balanced summary of what was done and what was found	1–2
**Introduction**			
Background/rationale	2	Explain the scientific background and rationale for the investigation being reported	2–4
Objectives	3	State specific objectives, including any prespecified hypotheses	4
**Methods**			
Study design	4	Present key elements of study design early in the paper	5
Setting	5	Describe the setting, locations, and relevant dates, including periods of recruitment, exposure, follow-up, and data collection	5–6, 8
Participants	6	(*a*) Give the eligibility criteria, and the sources and methods of selection of participants	5–6
Variables	7	Clearly define all outcomes, exposures, predictors, potential confounders, and effect modifiers. Give diagnostic criteria, if applicable	6–10
Data sources/ measurement	8*	For each variable of interest, give sources of data and details of methods of assessment (measurement). Describe comparability of assessment methods if there is more than one group	6–8
Bias	9	Describe any efforts to address potential sources of bias	9–10, 22–23
Study size	10	Explain how the study size was arrived at	5
Quantitative variables	11	Explain how quantitative variables were handled in the analyses. If applicable, describe which groupings were chosen and why	9–14
Statistical methods	12	(*a*) Describe all statistical methods, including those used to control for confounding	9–10
		(*b*) Describe any methods used to examine subgroups and interactions	12
		(*c*) Explain how missing data were addressed	9
		(*d*) If applicable, describe analytical methods taking account of sampling strategy	5, 9–10
		(*e*) Describe any sensitivity analyses	10–11, 18
**Results**			
Participants	13*	(a) Report numbers of individuals at each stage of study—e.g., numbers potentially eligible, examined for eligibility, confirmed eligible, included in the study, completing follow-up, and analyzed	11–12
		(b) Give reasons for non-participation at each stage	Not reported (no separate data collected on non-participants)
		(c) Consider use of a flow diagram	Not done (simple cross-sectional recruitment in two units)
Descriptive data	14*	(a) Give characteristics of study participants (e.g., demographic, clinical, social) and information on exposures and potential confounders	11–14; Tables 1–4
		(b) Indicate number of participants with missing data for each variable of interest	9 (overall missingness low; handling described; no per-variable counts)
Outcome data	15*	Report numbers of outcome events or summary measures	12–14; Tables 2–4
Main results	16	(*a*) Give unadjusted estimates and, if applicable, confounder-adjusted estimates and their precision (e.g., 95% confidence interval). Make clear which confounders were adjusted for and why they were included	15–17; Tables 5–6
		(*b*) Report category boundaries when continuous variables	11; Table 1

		were categorized	
		(*c*) If relevant, consider translating estimates of relative risk into absolute risk for a meaningful time period	Not applicable (no relative risk measures)
Other analyses	17	Report other analyses done—e.g., analyses of subgroups and interactions, and sensitivity analyses	10–13, 18
**Discussion**			
Key results	18	Summarize key results with reference to study objectives	18–19
Limitations	19	Discuss limitations of the study, taking into account sources of potential bias or imprecision. Discuss both direction and magnitude of any potential bias	22–23
Interpretation	20	Give a cautious overall interpretation of results considering objectives, limitations, multiplicity of analyses, results from similar studies, and other relevant evidence	18–22
Generalisability	21	Discuss the generalisability (external validity) of the study results	22–24
**Other information**			
Funding	22	Give the source of funding and the role of the funders for the present study and, if applicable, for the original study on which the present article is based	26

*Give information separately for exposed and unexposed groups.

Note: An Explanation and Elaboration article discusses each checklist item and gives methodological background and published examples of transparent reporting. The STROBE checklist is best used in conjunction with this article (freely available on the websites of PLoS Medicine at http://www.plosmedicine.org/, Annals of Internal Medicine at http://www.annals.org/, and Epidemiology at http://www.epidem.com/). Information on the STROBE Initiative is available at www.strobe-statement.org.

Appendix S2. Analytic procedures in IBM SPSS Statistics

All statistical analyses were conducted using IBM SPSS Statistics, version 28.0. The correlation matrix in Table 5 was generated with the Bivariate Correlations procedure (Analyze → Correlate → Bivariate), selecting Pearson coefficients (two-tailed), and additionally Spearman rank correlations for satisfaction as a sensitivity check given its skewed distribution. The 'Flag significant correlations' and 'Means and standard deviations' options were requested to facilitate interpretation of effect sizes and precision. The correlation coefficients, 95% confidence intervals, and p values reported in the manuscript were taken directly from this SPSS output, and spot-checks confirmed that all tabled values match the underlying output.

The multivariable linear regression model predicting total self-efficacy (SEMCD-6) was estimated using the Linear Regression procedure (Analyze → Regression → Linear). SEMCD-6 total score was entered as the dependent variable, with DSI total score, PSNCQQ total score, and pre-specified sociodemographic, clinical, and laboratory covariates as independent variables, entered simultaneously in a standard (enter) model. Under 'Statistics', the options for estimates, confidence intervals, model summary (including R, R^2, adjusted R^2), collinearity diagnostics, and ANOVA were selected. Standard diagnostic plots (standardized residuals vs. predicted values, normal probability plot) were requested to assess linearity, normality, and homoscedasticity of residuals. The unstandardized coefficients (B), standardized coefficients (β), 95% confidence intervals, adjusted R^2, F statistic, and model p value reported in Table 6 and the Results section were copied from the SPSS Coefficients, Model Summary, and ANOVA tables, and manual checks verified that all reported figures are consistent with the original SPSS output.

No additional data transformations were applied between SPSS output and table preparation, and all coefficients, confidence intervals, and model-fit statistics in Tables 5 and 6 were double-checked against the saved SPSS output files before submission.
